# Will top-ranked scientific publications become the preserve of “rich” groups? ASM editorial policy

**DOI:** 10.1128/mbio.03659-25

**Published:** 2026-03-09

**Authors:** Juan Carlos Argüelles

**Affiliations:** 1 Área de Microbiología, Facultad de Biología, Universidad de Murcia 98701 , Murcia, Spain; Georgia Institute of Technology, Atlanta, Georgia, USA

**Keywords:** scientific publications, ASM editorial policy, rich groups, article processing charges, open access

## LETTER

The extraordinary scientific revolution recorded at the beginning of the 20th century, best defined as a “change of paradigm” (1), was based on the acceptance of the universal value of science, implying that any new breakthrough must be demonstrated and be reproducible by anyone anywhere. One corollary of this was the need to establish a new, unifying, and supposedly objective procedure of scientific communication. The chosen path was to report original advances in short articles, written in a consensus language (English) and published in specialized journals to be accessible worldwide. Prior evaluation of an article and its approval by experts would guarantee the veracity and relevance of its scientific content. There were many vicissitudes in such a publication system, and the unexpected explosion of the internet marked a cutting-edge, almost Copernican change. Another amendment was the substitution of the lone researcher by hierarchically organized groups of investigators.

Present-day specialized journals are frequently part of large editorial corporations. Perhaps as a consequence, for many years, standard publications usually involve an important economic cost for the research group in question. A variety of formulas exist, ranging from personal or institutional subscription to article processing charges (APC). In an open access (OA) program, the costs of publication are paid directly by the authors or by the academic/scientific institutions where they work. Here, I analyze the editorial policy followed by the American Society for Microbiology (ASM) (2).

In 2025, the ASM established a new dual pathway for publication fees. (i) “Subscribe to open” is based on the subscriptions to six selected journals. In this approach, the ASM will set a minimum financial target each year, which, when reached, will render the content open access (2). (ii) Another group of journals is maintained within an OA formula, whereby the APC varies between ASM members and non-members and between various article types. Such fees are subject to change without notice (2). The ASM does not charge for certain types of publication (such as Editorials, Letters, and Perspectives). Likewise, the ASM has a policy of waivers, depending on specific evaluation (2).

## RICH AND POOR RESEARCH TEAMS

On the other hand, publications have other implications beyond scientific relevance itself. Active metric parameters (impact factor, citations, quartiles, etc.) attempt to judge research quality with apparent rigor and objectivity. This procedure is widely criticized, and its impartiality and flaws are questioned, including suggestions of fraud and plagiarism, which may lead to retraction (3–7). However, most public and private institutions have adopted this strategy as an essential criterion for their scientific policy—a worrying situation in countries like Spain which tend to give little value to scientific research (8).

Consequently, those selected as being “excellent research teams” receive generous financial support, enabling them to increase both human capital and equipment. These “privileged” groups publish their achievements in “top” journals, which involves high publication costs (a threshold of between $5,000 and $12,000). The journals’ visibility and prestige are also affected (9). Thus, some classic publications in microbiology which have recorded seminal breakthroughs are at risk of extinction (10). However, in this mercilessly competitive system, we should ask some questions: have fundamental advances always/only been published in top journals? Does the intense production of papers necessarily involve transcendental discoveries? And, more importantly, regarding the question addressed here…can it be assured that the articles proposed by distinct research teams are treated equally? I suggest that the answer is no. It seems indisputable that the current policy is benefiting well-financed teams (rich or privileged laboratories) to the detriment of those endowed with scarce resources (poor or deprived groups) who cannot afford journal charges. This situation does not represent a flat playing field and can only add to the distance between developed and developing countries, the latter being limited with regard to the financial support they receive to perform scientific research. The perspectives for the near future are certainly not promising. Conceivably, a parallel may be drawn with the phenomenon of social communication media.

## THE SEARCH FOR SOLUTIONS

Any proposals to find fair and suitable solutions are not immediately evident. However, some rational initiatives are in progress, and others might be implemented in the near future. For example, (i) many public and private institutions have established agreements with specific journals’ publishers to partially or totally cover the costs of publication; (ii) editorial rules should regulate the number of authors of a given paper; and (iii) variable charges could be afforded as a function of the financial support received by the research groups, which would imply honest and ethical behavior on their part. Alternatively, a progressive policy of waivers, discounts, and editorial help might be applied. The commitment of ASM is pioneering in this strategy, throughout its continuous effort to promoting equity, diversity, and inclusion as essential elements for the successful dissemination of microbial research.

A final reflection, perhaps of little significance, might be the application of tariffs as a function of the number of authors involved in each publication. It is frequent to find papers released by international cooperative consortia with the work “signed” by numerous authors. Indeed, I have recently revised the “Guidelines for monitoring autophagy,” in whose 4th edition 2,198 authors participated (11), all of them regarded as equal intellectual “fathers of the creature,” with the concomitant professional repercussions for research grants and/or academic posts. Hence, I wonder whether a specific APC should be established for each individual author, enabling small groups to obtain some modest compensation with respect to the “excellent” giant teams. It should be remembered that many scientific breakthroughs in microbiology and other disciplines were achieved by tenacious small groups and/or outstanding single individuals (12). Whatever the case, although the dramatic sentence “Publish or perish” lacks any satisfactory explanation, perhaps we should not forget that, according to Szent-Györgyi, “Research is what everyone sees, but no one has thought.”
